# The spread of the invasive mosquito *Aedes albopictus* (Diptera: Culicidae) in Portugal: a first genetic analysis

**DOI:** 10.1186/s13071-024-06460-w

**Published:** 2024-09-13

**Authors:** Líbia Zé-Zé, Inês Campos Freitas, Manuel Silva, Patrícia Soares, Maria João Alves, Hugo Costa Osório

**Affiliations:** 1 Centre for Vectors and Infectious Diseases Research (CEVDI), Department of Infectious Diseases, National Institute of Health Doctor Ricardo Jorge (INSA), Águas de Moura, Portugal; 2 Center for the Study of Animal Science (CECA), Institute for Agricultural and Agro-Alimentary Science and Technology (ICETA), University of Porto, Porto, Portugal; 3 NOVA National School of Public Health, Public Health Research Centre, Comprehensive Health Research Center, NOVA University Lisbon, Lisbon, Portugal; 4 Institute of Environmental Health of the Faculty of Medicine, University of Lisbon (ISAMB), Environment and Infectious Diseases Research Group, Lisbon, Portugal

**Keywords:** *Aedes albopictus*, Haplotypes, COX, Portugal

## Abstract

**Background:**

*Aedes albopictus*, commonly known as the Asian tiger mosquito, has become one of the most invasive mosquito species. Over the last 5 decades, it has been introduced and established in various tropical and temperate regions worldwide. First reported in Europe in 1979 in Albania and later in Italy in 1990, the species is now established in 13 European Union (EU)/European Economic Area (EEA) countries and 337 regions (2023). In Portugal, *Ae. albopictus* was first detected in the Algarve and Penafiel regions in 2017, followed by Alentejo in 2022 and Lisbon in 2023. This mosquito species poses a significant public health risk as a vector for numerous pathogenic viruses, including dengue, Zika, and chikungunya.

**Methods:**

*Aedes albopictus* collected in Lisbon in 2023 were analyzed using *cytochrome c oxidase I* (*COX*) gene sequencing to understand their genetic relationships.

**Results:**

Our data indicate that the *Ae. albopictus* mosquito populations detected in three locations in Lisbon in 2023 correspond to recent but distinct introduction events.

**Conclusions:**

Although there has been no local transmission of *Aedes*-transmitted viruses in mainland Portugal to date, the spread of the mosquito and increased international travel increase the risk of *Aedes*-borne disease outbreaks. The ongoing spread of *Ae. albopictus* in the country and the confirmed multiple introductions in new locations raise awareness of the need to monitor mosquito vectors to control and prevent autochthonous *Aedes*-borne disease outbreaks.

**Graphical Abstract:**

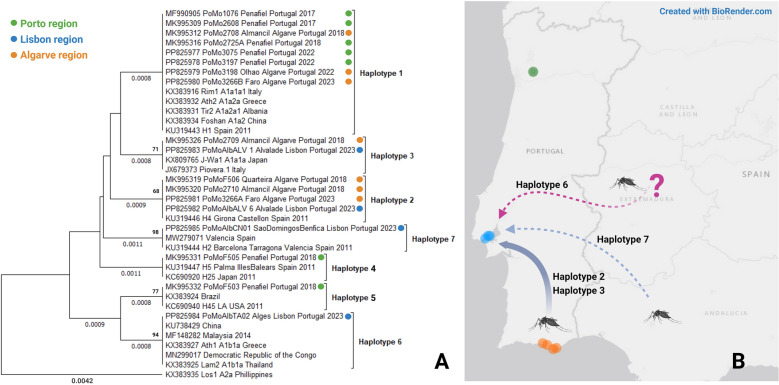

## Background

First described by Skuse in India in 1894, *Aedes (Stegomya) albopictus* has been recognized as one of the most invasive mosquito species, successfully colonizing numerous tropical and temperate regions worldwide over the last 5 decades. *Aedes albopictus* was first reported in Europe in 1979 in Albania [1], followed by Italy in 1990 [2]. Italy is currently considered the most infested country in Europe, with *Ae. albopictus* established over large areas and thriving particularly in urban areas [3]. Since its introduction in Italy, *Ae. albopictus* has steadily spread throughout Europe, particularly to most Mediterranean countries. In 2023, *Ae. albopictus* was established in 13 European Union (EU)/European Economic Area (EEA) countries and 337 regions, while in 2013, it was established only in 8 countries and 114 European regions [4]. In Portugal, *Ae. albopictus* was first detected in 2017 through two different introduction events in the Algarve, the southernmost region [5], and Penafiel, in the Porto region [6]. In 2022, this vector was detected in the Alentejo region and, in late, September 2023 in Lisbon [7]. The National Vector Surveillance Network-Rede de Vigilância de Vectores (REVIVE) has been running since 2008 under the auspices of the Portuguese Ministry of Health [8]. REVIVE conducts nationwide surveillance of the most critical hematophagous arthropods for public health (mosquitoes, ticks, and sandflies). Regular surveillance of mosquito species and screening field-collected mosquitoes for arboviruses is conducted. Airports, ports, storage areas, and certain border regions with Spain are monitored throughout the year with the involvement of local and regional authorities.

In addition to the nuisance associated with establishing *Aedes albopictus*, its ability to act as a vector for a wide range of arboviruses remains a primary concern. This mosquito species has emerged as a significant global public health threat due to its ability to transmit several pathogenic flaviviruses (such as dengue, Zika, and yellow fever) and alphaviruses (especially chikungunya virus). The central tourist regions in the country are the Algarve, Lisbon, and Porto, where the presence of this vector species can be a significant concern due to the higher risk of incoming viremic travelers. Since 2007, cases of autochthonous transmission of chikungunya associated with *Ae. albopictus* have been documented in Europe [9]. Dengue has been reported in Europe since 2010, with autochthonous cases transmitted by *Ae. albopictus* in Croatia, France, Italy, and Spain [10].

To date, local transmission of *Aedes*-borne viruses has not yet been detected in mainland Portugal. However, the geographic expansion of *Ae. albopictus* across Portugal, combined with the increasing number of international travelers, often from regions with ongoing *Aedes*-borne outbreaks, highlights the importance of mosquito vector surveillance and control and raises public health concerns about the risk of increased introduction and autochthonous transmission of *Aedes*-borne viral infections. Here, we report a preliminary genetic analysis of the *Ae. albopictus* mosquitoes detected in Lisbon in 2023, using the primary barcode sequence for members of the animal kingdom, a partial sequence of the *cytochrome c oxidase I* (*COX*) gene, widely used to study the genetic relationships of *Ae. albopictus* [11–14] and previously used in Portuguese mosquito populations [15].

## Methods

### Mosquito samples and DNA extraction

Larvae samples were collected in natural and artificial water containers at three sites in the Lisbon region (Algés, Alvalade, and São Domingos de Benfica; Table 1). Mosquito samples from the Algarve and Porto populations, collected in 2022 and 2023, were also submitted for analysis. All mosquito samples were collected by the national REVIVE surveillance network [6] in public and private properties, with the knowledge and permission of the respective responsible/owners. The collected mosquitoes were reared to adults in the insectary (by collection site and date), and 10 adult mosquitoes were randomly selected from each collection site for analysis. Sampled mosquitoes collected in the Lisbon region were ground individually by grinding with a mortar and pestle with liquid nitrogen and further ground after adding 500 µL lysis buffer (NUCLISENS® easyMAG, Biomérieux). Nucleic acid extraction was performed with the prepared lysate suspensions in the automated platform NUCLISENS® easyMAG (Biomérieux), as previously described [6]. Larval and adult mosquitoes were morphologically confirmed as *Ae. albopictus* [16, 17].

**Table 1:** *Aedes albopictus* samples collected in Portugal and included in this study

Original designation	*COX* GenBank ID	Collection date	Collection place	Region	Number of mosquitoes	*COX* haplotype	References
PoMo1076	MF990905	4 September 2017	Penafiel	Porto	1	1	[6]
PoMo2608	MK995309	12 September 2017	Penafiel	Porto	1	1	[15]
PoMo2725A	MK995316	17 October 2018	Penafiel	Porto	1	1	[15]
PoMo3075	PP825977	01 June 2022	Penafiel	Porto	1	1	This study
PoMo3197	PP825978	28 September 2022	Penafiel	Porto	7	1	This study
PoMoF505	MK995331	11 July 2018	Penafiel	Porto	1	4	[15]
PoMoF503	MK995332	11 July 2018	Penafiel	Porto	1	5	[15]
PoMo2708	MK995312	27 September 2018	Almancil	The Algarve	1	1	[15]
PoMo3198	PP825979	19 September 2022	Olhão	The Algarve	6	1	This study
PoMo3266B	PP825980	08 August 2023	Faro	The Algarve	6	1	This study
PoMoF506	MK995319	12 July 2018	Quarteira	The Algarve	1	2	[15]
PoMo2710	MK995320	26 September 2018	Almancil	The Algarve	1	2	[15]
PoMo3266A	PP825981	08 August 2023	Faro	The Algarve	6	2	This study
PoMo2709	MK995326	27 September 2018	Almancil	The Algarve	3	3	[15]
PoMoAlbALV_6	PP825982	26 October 2023	Alvalade	Lisbon	1	2	This study
PoMoAlbALV_1	PP825983	26 October 2023	Alvalade	Lisbon	9^a^	2	This study
PoMoAlbTA02	PP825984	26 October 2023	Algés	Lisbon	10^a^	6	This study
PoMoAlbCN01	PP825985	26 October 2023	São Domingos de Benfica	Lisbon	10^a^	7	This study

^a^Number of individually analyzed mosquitoes from the same collection site and date with identical *COX* sequence

### Molecular analysis

Molecular identification was performed using the *COX* gene of mitochondrial DNA with primers LCO1490 and HCO 2198 [18], as previously described [6]. *Aedes albopictus* haplotype diversity for *COX* sequences was estimated using DnaSP v.6.10.01 [19] using default parameters.

**Fig. 1:**
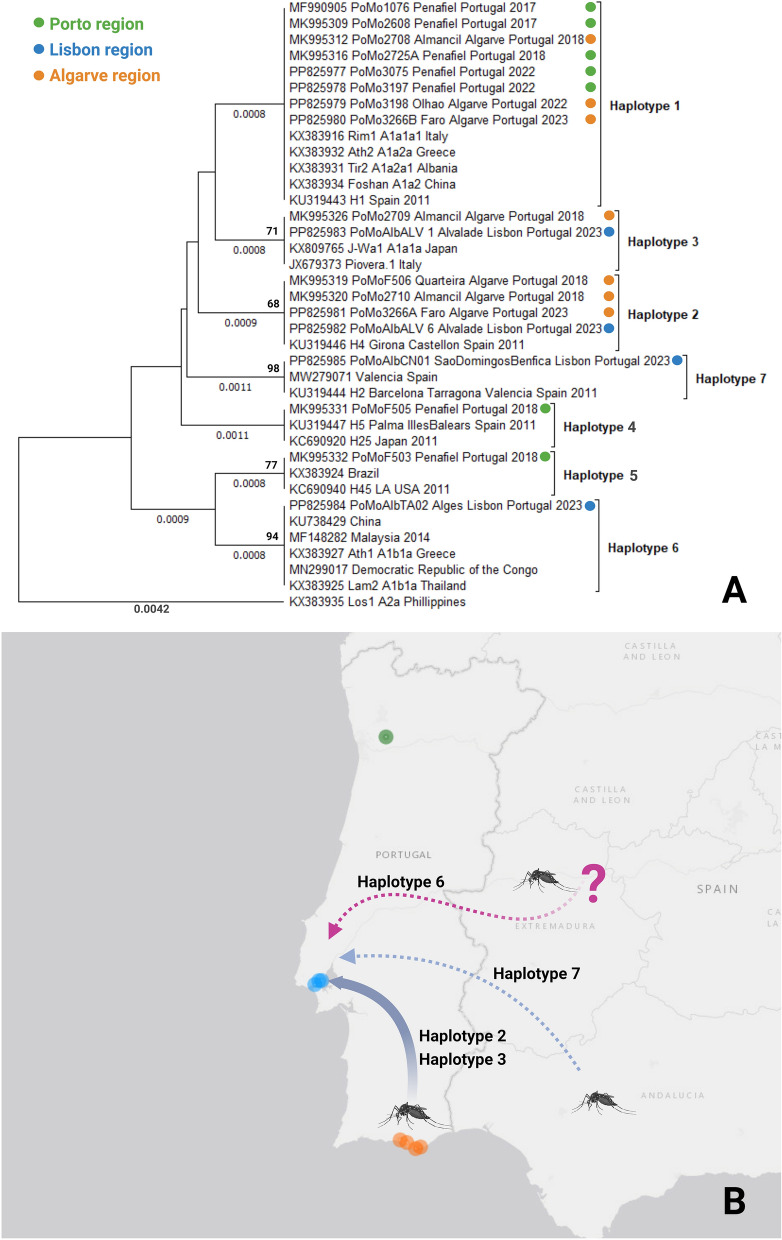
**A** UPGMA phylogenetic tree constructed from 18 (9 novel) *COX* sequences obtained from mosquitoes circulating in Portugal (Table 1) and 20 sequences available in GenBank. Bootstrap values (1000 replicates) greater than 65 are shown above the branches, and branch lengths (in the same units as those of the evolutionary distances used to infer the phylogenetic tree) are shown below the branches. Evolutionary distances were calculated using maximum composite likelihood. Sequences are identified by GenBank accession numbers, country region, country, and year of collection (if available). Tree nodes are identified by the *COX* haplotype. Colored circles indicate the regions where mosquitoes from Portugal were collected. **B** Map showing the locations of mosquito sample collection and the most likely origin of introduction in Lisbon, based on available data. Composite figure created with https://www.biorender.com/

To integrate the mosquitoes circulating in Portugal and into the global *Ae. albopictus* genetic diversity, the consensus nucleotide partial sequences of the *COX* gene were aligned against several sequences available in GenBank (20 *COX* previously reported at the global level; Fig. 1) using BioEdit version 7.2.5 [20] and further used to construct a phylogenetic tree using the unweighted pair group method with arithmetic mean (UPGMA; 1000 bootstraps) in MEGA X [21]. For better visualization of the tree, the KX383935 sequence was used as an outgroup. Figure 1 was generated using BioRender.com.

## Results

The 10 partial *COX* sequences obtained for *Ae. albopictus* mosquitoes collected at each of the three sites in Lisbon (30 sequences in total) show low genetic diversity, with only one haplotype in Algés (GenBank accession number PP825984) and São Domingos de Benfica (GenBank accession number PP825985). In Alvalade, two haplotypes were identified, with one corresponding to nine collected mosquito specimens (GenBank accession number PP825983) and the other corresponding to only one (GenBank accession number PP825982). Sequences from mosquitoes collected in 2022 and 2023 in the Algarve and Porto regions are identical to previously detected haplotypes for these regions (Table 1; Fig. 1), which were identified as haplotype 1 (the Algarve and Porto) and haplotype 2 for the Algarve, as previously defined [15]. The haplotype diversity analysis for the sequences detected in the Lisbon region shows, in Alvalade, two haplotypes, haplotype 2 and haplotype 3, previously detected in the Algarve, and two new haplotypes for the other sites, haplotype 6 in Algés (with similarity to sequences from widespread sites) and haplotype 7 in São Domingos de Benfica, identical to sequences detected in Spain (Table 1 and Fig. 1).

## Discussion

The observed low diversity of *COX* sequences in mosquitoes collected in Lisbon is consistent with recent introductions. Although the collection of immature mosquitoes may bias the population diversity, collections were made in heavily infested containers, corresponding to multiple female mosquito ovipositions. For most of these sites, the water containers containing immature mosquitoes were the only ones found with *Ae. albopictus* in the surveyed area. Some sites were analyzed following citizen science reports of mosquito presence, and others resulted from detailed inspections by the Lisbon and Tagus Valley Health Authority following the initial detection event.

The *COX* sequences from the three Lisbon sites differed, so these introductions can be considered separate events. Although preliminary, these data suggest that, at least in Alvalade, the introduction may have come from the Algarve. However, direct external introductions cannot be excluded, especially in São Domingos de Benfica and Algés. Nevertheless, more data are needed on the current mosquito populations circulating in Porto, the Algarve, and Alentejo. A finer genetic analysis, namely by mitogenome sequencing, with a broader sampling of mosquitoes in the coming season (2024) is underway to obtain more details. Nevertheless, the identification of three independent introduction events in one mosquito season highlights the potential of this species to invade new geographic areas in a short period. It also means that these events are likely underway and indicate naive regions that could be rapidly colonized.

## Conclusion

In 2022, over 19 million travelers entered Europe from dengue-affected areas [22]. Between 2012 and 2022, our team at the National Reference Laboratory of the Portuguese National Institute of Health (INSA) detected 142 *Aedes*-borne infections in viremic travelers. However, the high rates of asymptomatic infection in humans and the relatively short viremic window of symptomatic patients suggest that many traveler infections may be under-recognized, and the number of viremic travelers is much higher. Europe is experiencing a warming trend, with more frequent and severe heat waves and floods and longer and warmer summers [10]. This creates more favorable conditions for invasive mosquito species such as *Ae. albopictus* and *Ae. aegypti*. The geographical spread of invasive mosquito species to previously unaffected areas in the EU/EEA is an ongoing reality. In most European countries, cold winters do not allow year-round transmission [10], but in the southernmost region of Portugal, the Algarve, adult *Ae. albopictus* mosquitoes are already present year-round, although at lower population levels in winter. Given climate change, with continued increases in temperature and subsequent milder winters, the conditions for virus transmission will undoubtedly increase.

Given the ongoing spread of *Ae. albopictus* in mainland Portugal, it is essential to raise awareness of mosquito-borne diseases among the general public, healthcare professionals, and travelers.

## Data Availability

The nucleotide sequence data reported in this paper have been deposited in the NBCI GenBank database under the accession numbers: PP825977–PP825985.

## Abbreviations

*COX*: *Cytochrome c oxidase I*
EU/EEA: European Union/European Economic Area
FCT: Portuguese Foundation for Science and Technology
